# Resistance Exercise Training as a New Trend in Alzheimer’s Disease Research: From Molecular Mechanisms to Prevention

**DOI:** 10.3390/ijms25137084

**Published:** 2024-06-27

**Authors:** Alexis Sepúlveda-Lara, Paulina Sepúlveda, Gabriel Nasri Marzuca-Nassr

**Affiliations:** 1 Doctorado en Ciencias mención Biología Celular y Molecular Aplicada, Facultad de Ciencias Agropecuarias, Universidad de La Frontera, Temuco 4811230, Chile; a.sepulveda23@ufromail.cl; 2 Departamento de Ciencias Preclínicas, Facultad de Medicina, Universidad de La Frontera, Temuco 4811230, Chile; paulina.sepulveda@ufrontera.cl; 3 Departamento de Ciencias de la Rehabilitación, Facultad de Medicina, Universidad de la Frontera, Temuco 4811230, Chile

**Keywords:** Alzheimer’s disease, neurodegenerative diseases, aging, resistance exercise training, exercise

## Abstract

Alzheimer’s disease is a pathology characterized by the progressive loss of neuronal connections, which leads to gray matter atrophy in the brain. Alzheimer’s disease is the most prevalent type of dementia and has been classified into two types, early onset, which has been associated with genetic factors, and late onset, which has been associated with environmental factors. One of the greatest challenges regarding Alzheimer’s disease is the high economic cost involved, which is why the number of studies aimed at prevention and treatment have increased. One possible approach is the use of resistance exercise training, given that it has been shown to have neuroprotective effects associated with Alzheimer’s disease, such as increasing cortical and hippocampal volume, improving neuroplasticity, and promoting cognitive function throughout the life cycle. However, how resistance exercise training specifically prevents or ameliorates Alzheimer’s disease has not been fully characterized. Therefore, the aim of this review was to identify the molecular basis by which resistance exercise training could prevent or treat Alzheimer’s disease.

## 1. Introduction

Alzheimer’s disease (AD) is a neurodegenerative disease characterized by the progressive loss of neuronal connections. It is the most common type of dementia in elderly people [1]. At present, there are approximately 50 million people with AD throughout the world; this number is expected rise to 152 million by 2050 [2]. AD is a priority pathology for public health. It has been described as the greatest global health challenge of the 21st century, given the increase in life expectancy, which has increased the incidence and prevalence of AD [3]. A prominent challenge regarding AD is the ethical, social, and political pressure on the allocation of health and research resources, due to the high costs of diagnosis and treatment, as well as the high impact of this pathology on the quality of life of patients and their caregivers [4]. One of the main complications in the treatment of AD is the lack of an effective method to diagnose patients: a confirmation of AD is only postmortem [5]. Therefore, there have been intensive effectors aimed at prevention. Resistance exercise training (RET), also called strength training, has been proposed as a non-pharmacological preventive therapy, because it improves working memory in children [6], adults [7], and older people [8,9].

There has been an exponential increase in studies that seek to prevent, diagnose, and treat AD. RET is a possible alternative to prevent or treat AD due to its numerous effects. It increases brain-derived neurotrophic factor (BDNF) levels, which improve neuroplasticity and cognitive function [10]. Moreover, moderate-to-high-intensity general physical activity has been shown to increase hippocampal volume in older adults with AD after a 24-month intervention program [11]. Additionally, recent studies suggest that RET can reduce the levels of proinflammatory cytokines, such as tumor necrosis factor alpha (TNF-α) and interleukin 6 (IL-6), and increase the levels of anti-inflammatory cytokines, such as IL-10. These effects may contribute to reducing neuroinflammation and to promoting a more favorable brain environment for neuronal function and cell survival in animal and human models [12,13,14]. In animal models, RET has demonstrated its ability to promote the clearance of beta-amyloid (Aβ) [15,16,17], as well as to reduce both the volume and number of Aβ plaques [14,18]. RET also decreased intracellular neurofibrillary tangles (NFTs) in mouse brain [14]. Furthermore, researchers have postulated that RET induces an increase in glutamate release, stimulating the N-methyl-D-aspartate receptors (NMDARs). This activation increases calcium cations (Ca^2+^) in postsynaptic neurons, triggering the activation of calcium calmodulin kinase II (CaMKII), followed by the mitogen-activated protein kinase (MAPK)/extracellular signal-regulated kinase (ERK) signaling pathway and the transcription factor cAMP responsive element bindings protein 1 (CREB1) that modulate BDNF [19]. These findings support the use of RET as a potential preventive therapy for AD by modulating BDNF. While the use of RET as a strategy to prevent or treat AD has yielded promising results in animal models, as of March 2024, ClinicalTrials.gov includes relatively few studies that have utilized RET as an intervention in older individuals diagnosed with AD. Most of these studies have been published within the last 5 years and mention characteristics related to general or aerobic physical exercise.

## 2. Alzheimer’s Disease

Generally, AD is classified into two forms: familial and sporadic. The familial form represents 1–5% of the total cases and is categorized as early-onset AD (EOAD) because it generally occurs in patients younger than 65 years. This form presents genetic mutations in presenilin 1 (PSEN1), presenilin 2 (PSEN2), or amyloid precursor protein (APP). On the other hand, sporadic AD represents 95% of cases and is categorized as late-onset AD (LOAD), occurring in patients older than 65 years. Several risk factors have been identified in sporadic AD, with aging being the main one [20].

Both forms of AD have a similar phenotype associated with neuronal death due to genetic, epigenetic, cerebrovascular, and environmental factors [21]. In addition, both EOAD and LOAD present the same mechanism of action and pathophysiology, which is centered on three hypotheses that have been duly tested and supported: the amyloid, cholinergic, and tau hypotheses of neuroinflammation and neurotoxicity. Atypical clinical presentations for AD can also occur, and they have garnered increased interest in the past few decades, with research now focusing on investigating the underlying mechanisms and improving the diagnosis of these atypical AD presentations [22]. However, their extent, frequency of overlap, and neuroanatomical underpinnings remain unclear [23]. Cases of typical AD usually begin in the entorhinal cortex within the hippocampus [24], while atypical AD is characterized by the onset of neurodegeneration in cortical areas, leading to posterior cortical atrophy [25] and frontotemporal atrophy [26]. Other atypical forms include the logopenic variant, primary progressive aphasia, and amnestic syndrome with multidomain impairment [27].

### 2.1. Main Hypotheses for Alzheimer’s Disease

**Figure 1:**
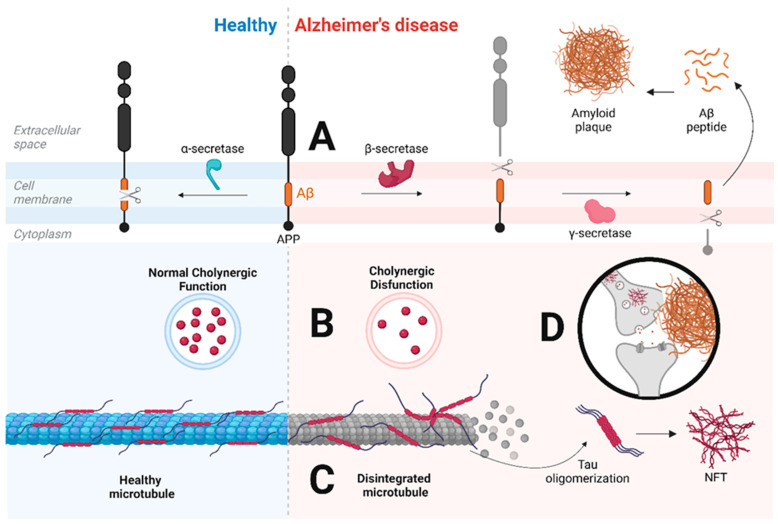
Comparison of the molecular mechanism of a healthy neuron (**left**) versus a neuron with Alzheimer’s disease (AD) (**right**). (**A**) shows normal APP cleavage, where α-secretase cleaves APP into Aβ peptide. However, when this cleavage is disrupted, a first cleavage is carried out by β-secretase, followed by a second cleavage by γ-secretase, which releases Aβ into the extracellular space, where it oligomerizes to form amyloid plaque. (**B**) shows a difference in cholinergic function: the neuron with AD presents a decrease in cholinergic activity due to decreased acetylcholine. (**C**) shows how, in the neuron with AD, there is disintegration of microtubules due to tau hyperphosphorylation. This hyperphosphorylation leads to tau oligomerization and the formation of NFTs in the intracellular space. Finally, (**D**) exemplifies the dysfunctionality of the connection between cholinergic neurons. Abbreviations—Aβ = amyloid beta; APP = amyloid precursor protein; NFT = neurofibrillary tangle. This figure was created at https://app.biorender.com on 15 June 2023.

The amyloid hypothesis (Figure 1A) describes the formation of amyloid plaques in extracellular space, also called senile plaques, which are produced by an alteration in the cleavage of APP [28]. The cholinergic hypothesis (Figure 1B) proposes the symptomatology of AD results from a decrease in cholinergic function due to a decrease in the neurotransmitter acetylcholine, both in its production and uptake by the postsynaptic neuron, which are caused by microtubule disintegration and obstruction from senile plaques, respectively [29]. The tau hypothesis (Figure 1C) describes the formation of NFTs as a result of tau hyperphosphorylation, which degrades the microtubules and impairs the chemical transmission of the neuron, including internal transport [30]. Neurodegeneration is triggered due to the accumulation of senile plaques and NFTs (Figure 1D).

### 2.2. Other Hypotheses for Alzheimer’s Disease

**Figure 2:**
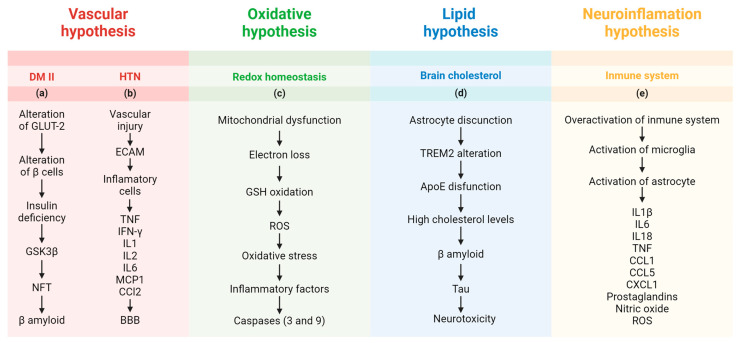
This representative figure summarizes the molecular mechanisms and new hypotheses showing how diabetes mellitus, arterial hypertension, oxidative stress, and brain cholesterol could lead to Alzheimer’s disease. Abbreviations—ApoE = apolipoprotein E; BBB = blood–brain barrier; CCL = chemokine (C-C motif) ligand; CXCL = chemokine (C-X-C motif) ligand; DM II = type 2 diabetes mellitus; ECAM = endothelial cell adhesion molecule; GLUT-2 = glucose transporter 2; GSH = glutathione; GSK3β = glycogen synthase kinase 3 beta; IFN- γ = interferon gamma; IL = interleukin; MCP1 = monocyte chemoattractant protein 1; NFT = neurofibrillary tangle; TNF = tumor necrosis factor; TREM2 = triggering receptor expressed on myeloid cells 2. This figure was created at https://app.biorender.com on 21 July 2023.

Other, newer hypotheses have been proposed to explain the pathophysiology of AD (Figure 2), with the aim of finding new molecular mechanisms that allow a better understanding of AD.

#### 2.2.1. Vascular Hypothesis

The vascular hypothesis (Figure 2 (a)), which is related to cardiovascular diseases [31], has two general approaches that seek to describe the mechanism of action leading to AD. The first approach is provoked by diabetes mellitus. An altered glucose metabolism has been reported in patients with AD [32]. This phenomenon has been evaluated in cell and animal models by using streptozotocin. When administered peripherally, this drug induces diabetes, cytotoxicity, oxidative stress, and mitochondrial dysfunction [33]. Streptozotocin damage pancreatic β-cells, affecting glucose transporter 2 (GLUT-2), increasing insulin deficiency, and promoting hyperglycemia [34]. When streptozotocin is injected into the brain, it leads to cognitive impairment and pathophysiological alterations like those found in patients with AD, including cerebral insulin resistance, oxidative stress, tau phosphorylation, and neuroinflammation [35,36]. Diabetes mellitus was one of the first diseases studied in relation to the molecular effects of RET on health and disease [37]. Specifically, RET prevents and even ameliorates diabetes mellitus by recruiting and translocating GLUT-4, thus counteracting insulin resistance in patients with type 2 diabetes [37].

Another focus of the vascular hypothesis is arterial hypertension (Figure 2 (b)), given the close relationship between this disease and the prevalence of AD [38]. In general, these associations have been studied with physiological markers such as blood pressure, alongside imaging. Researchers have identified higher levels of Aβ and tau bodies in the brains of patients with hypertension [39,40]. It has been postulated that vascular injury increases the expression of adhesion molecules by endothelial cells, facilitating the recruitment of inflammatory cells, including lymphocytes, macrophages, and monocytes, which are responsible for secreting proinflammatory mediators such as TNF, interferon-γ, IL-1, IL-2, IL-6, and monocyte chemoattractant protein 1 (MCP1, also known as chemokine (C-C motif) ligand 2 [CCL2] [41]. These changes alter the blood–brain barrier and promote neurotoxicity.

#### 2.2.2. Oxidative Hypothesis

The oxidative hypothesis, also called the mitochondrial cascade hypothesis (Figure 2 (c)), considers the mitochondrial function of neurons [42,43], specifically the oxygen requirements of the brain and how metabolic abnormalities could lead to AD [44,45]. Mitochondrial dysfunction and damage could increase oxidative stress; inhibit mitochondrial functions in cells such as astrocytes; and hinder brain bioenergetics, neurotransmission, and redox balance in astrocytes [46]. This oxidative stress is caused by reactive oxygen species (ROS) through the loss of electrons from the electron transport chain in complexes I, II, III, IV, and V. ROS oxidizes mitochondrial glutathione (GSH) and alters intramitochondrial redox homeostasis, leading to irreversible oxidative modifications of mitochondrial DNA [47]. Consistently, it has been confirmed that excessive mitochondrial ROS production can activate inflammatory factors that trigger apoptosis through the activation of caspase-9 and caspase-3 [48,49]. In addition to the excessive release of ROS, the hypothesis states that a progressive decline in mitochondrial fitness and adenosine triphosphate (ATP) production are significant contributors to the pathological aggregation of proteins, reduced synaptic plasticity, and cognitive impairment in AD [50]. Neurons are mostly oxidative, and glial cells process glucose via glycolysis, with the astrocyte–neuron lactate shuttle metabolically coupling the different cell types [51]. However, therapies targeting mitochondria in AD, whether oxidative or antioxidative, have not been successful so far, and innovative options such as mitochondria-derived mechanisms from hibernation may provide valuable new insights [50].

#### 2.2.3. Lipid Hypothesis

The lipid hypothesis (Figure 2 (d)) relates cholesterol metabolism and APP. This protein controls the turnover of cholesterol necessary for neuronal activity. It regulates the activity of oxysterols, to stimulate the synthesis of transporters involved in the exchange of amyloid peptides between the blood and the brain. The results altered Aβ production regulated by cholesterol and/or APP via lipid rafts [51,52,53]. In the brain, astrocytes regulate cholesterol. These cells provide metabolic support to neurons. They not only provide metabolic substances but can also metabolically suppress neuronal cholesterol synthesis and promote neuronal functions through secreted effector molecules, such as triggering receptor expressed on myeloid cells 2 (TREM2) and apolipoprotein E (ApoE) [54,55]. ApoE4 overexpression in pericytes induces cerebral amyloid angiopathy and leads to the contraction of endothelial cells, decreasing the tension of tight junction networks, which results in the loss of blood–brain barrier integrity [56]. Aging results in a decrease in the biosynthesis of these proteins, whose attrition leads to the dysregulation of cholesterol metabolism, which is hypothesized to increase the release of Aβ and tau, triggering neurotoxicity. Additionally, L5, the most electronegative subfraction of low-density lipoprotein (LDL), could be a crucial factor in understanding the involvement of lipids in AD pathology. Indeed, lectin-type oxidized LDL receptor 1 (LOX-1), one of the receptors involved in the internalization of L5, could trigger apoptotic pathways [57].

#### 2.2.4. Neuroinflammation Hypothesis

The neuroinflammation or neuroimmunomodulation hypothesis (Figure 2 (e)) suggests that chronic inflammation and dysregulation of the immune system in the brain play a key role in the progression of AD [58,59,60,61]. This hypothesis posits that the overactivation of the immune system leads to the activation of microglia and astrocytes, releasing proinflammatory cytokines, including IL-1β, IL-6, IL-18, and TNF; chemokines such asCCL1, CCL5, and chemokine (C-X-C motif) ligand 1 (CXCL1); prostaglandins; nitric oxide; and ROS [62]. These inflammatory mediators contribute to neuronal damage and death, potentially accelerating the accumulation of amyloid plaques and NFTs, which are hallmark features of AD [63]. For example, overactivation of the Cdk5/p35 pathway in microglia can lead to excessive release of proinflammatory and toxic products such as IL-1β and IL-6, as well as ROS. These inflammatory mediators can increase the production of Aβ and exacerbate tau hyperphosphorylation, triggering AD [64].

## 3. Resistance Exercise Training

It is necessary to understand the differences between physical activity and physical exercise to understand RET. Physical activity is any movement that involves skeletal muscle energy expenditure [65]; however, in this review we define physical activity as any action that involves sustained (isometric) or repetitive (isotonic) muscle contraction over time. On the other hand, physical exercise is a subset of planned, structured, and repetitive physical activity aimed at improving or maintaining physical fitness [66]. Based on this definition, physical exercise can be subdivided into various types of training, for example aerobic and resistance training, which is the focus of this review. RET, also called strength training, is a form of periodic physical exercise that provides a progressive overload on the contraction of skeletal muscles using external weights [67], thus stimulating their strength and mass, allowing them to lift more weight, and generating an increase in their size (hypertrophy).

### 3.1. Molecular Mechanisms

RET promotes skeletal muscle protein synthesis, which allows for strength and hypertrophy gains through the activation of the mammalian target of rapamycin complex 1 (mTORC1) pathway [68]. In addition, the phosphorylation of p70 (S6K), a downstream target of mTORC1, is strongly related to muscle hypertrophy [69]. Events associated with the upregulation of eukaryotic translation initiation factor 2B (eIF2B) may orchestrate the acute changes in protein synthesis after RET, while the activation of mTOR/S6k may result in preferential synthesis of proteins required to enhance the translation apparatus and to optimize long-lasting protein synthesis [70]. It is now known that increasing the skeletal muscle working weight load increases some hypertrophy-induced targets such as insulin-like growth factor 1 (IGF1), leading to an increase in muscle mass by stimulating phosphatidylinositol-3 kinase (PI3K) and RAC-alpha serine/threonine-protein kinase (Akt). This signaling pathway results in the subsequent activation of targets that are necessary for the synthesis of proteins such as mTORC1 [71,72,73,74].

### 3.2. Considerations

Among the considerations in a RET intervention are the four principles of training: overload, specificity, progression, and reversibility. Overload tries to provide an adequate stimulus to provoke the desired adaptation, whether physical, physiological, or in performance; to achieve an adequate overload, training must have an adequate intensity (percentage of maximum strength), frequency, and duration (total volume of training) [75]. Specificity considers the biomechanics and bioenergetics of the exercises to be used. The highest degree of performance adaptation that can be achieved from an exercise is sought, and the most appropriate is chosen depending on the proposed objective, which may be a maximum strength gain or an improved rate of force development, among others [75]. Progression is the gradual and systematic increase in the stimulus provided during training, referring to the stress to maintain the overload of skeletal muscle, generating a continuous adaptation from training [76]. Finally, reversibility is the observation or reminder that skeletal muscle disuse causes skeletal muscle deterioration, leading to a loss of training adaptations, indicating the importance of not only being physically active, but also maintaining an adequate overload stimulus.

## 4. Resistance Exercise Training as a Preventive Strategy for Alzheimer’s Disease

### 4.1. Alzheimer’s Disease in Skeletal Muscle and Brain

AD research has conventionally focused on the central nervous system, but it is now understood that various peripheral and systemic abnormalities are related to this disease [77]. Indeed, in AD mice, there is a relationship between skeletal muscle and AD, where hemopexin, the plasma protein responsible for transporting and eliminating the breakdown products of free hemoglobin to the cells of the reticuloendothelial system, is induced by skeletal muscle atrophy, leading to accelerated cognitive impairment [78]. In addition, abnormal APP metabolism with Aβ deposition has been demonstrated in the skeletal muscle tissue of mice [79]. These findings suggest that there are common pathological processes between the brain and skeletal muscle in patients with AD. Moreover, patients with dementia may have a higher prevalence of frailty and sarcopenia [80], which is a pathology characterized by the loss of skeletal muscle mass, strength, and function. Since 1984, it has been postulated that abnormal weight loss and cachexia are clinical criteria for the diagnosis of AD [81]. This was reinforced in 2008 with the proposal of the inclusion of body myositis, a pathology of muscle fibers whose molecular mechanism resembles what occurs in AD brains [82]. In myositis, IL-6B leads to the accumulation of Aβ through nitric oxide stress in vitro, reinforcing the relationship between skeletal muscle and AD [83]. At present, this is not part of the clinical diagnosis; rather, it is considered a risk factor for AD because the loss of lean mass is accelerated in AD, associating skeletal muscle atrophy with brain atrophy and cognitive impairment in individuals without dementia [84]. The molecular mechanisms are still unclear, but the skeletal muscle of patients with AD may be more prone to oxidative and inflammatory stress [85]. RET has shown beneficial effects in animal models of AD by promoting the clearance of Aβ, reducing Aβ plaques, and decreasing tau pathology in the brain [14,17,18]. The proposed mechanisms for this effect include the negative regulation of Aβ-forming enzymes, modulation of microglial activity, and reduction of astrogliosis [12,13,14]. Finally, it has been shown that there is a close relationship between skeletal muscle atrophy and brain atrophy in patients with AD [86], which strengthens the relationship between skeletal muscle atrophy and brain atrophy in AD. Taken together, RET may represent a possible non-pharmacological preventive therapy.

### 4.2. Resistance Exercise Training and Main Hypotheses for Alzheimer’s Disease

RET is proposed as a possible preventive therapy for AD by modulating mTORC1 and BDNF, two proteins that have been shown to influence the pathophysiology of AD. The PI3K/Akt/mTORC1 signaling pathway seems to be reduced in AD [87]. Strategies to increase this signaling have been sought with compounds such as suramin [88]. Indeed, enhancing the PI3K/Akt/mTORC1 signaling pathway improved deficits in hippocampal-dependent memory and activity-dependent synaptic protein synthesis in an AD mouse model [89]. On the other hand, increasing the BDNF concentration in the brains of double transgenic APP/PS1 mice improved cognitive function via the ApoE mimetic peptide COF1410, resulting in reduced Aβ deposition [90,91]. Furthermore, physical exercise in general has shown beneficial effects on BDNF, which may be involved in protecting the dynamics of myelin deficits in mouse models of AD [92]. In addition, BDNF has also been proposed as a potential biomarker due to its high implication in the molecular biology of AD [93,94].

**Figure 3:**
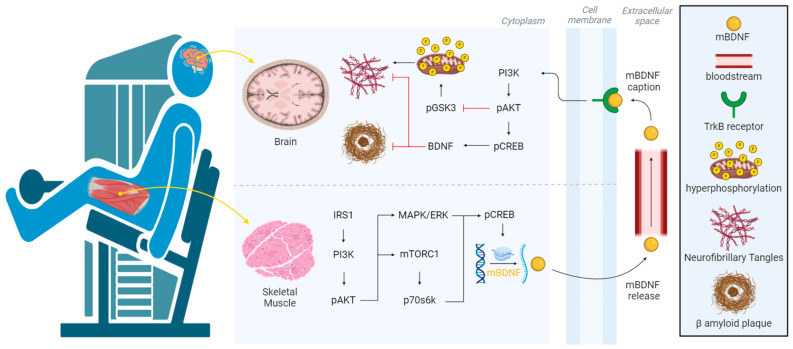
Visualization of how skeletal muscle contraction activates the PI3K/AKT, MEK/ERK, or mTORC1 pathway to promoting the transcription of BDNF. After cleavage, mBDNF is released into the bloodstream. Subsequently, mBDNF crosses the blood–brain barrier and binds to TrkB on the neuronal surface, activating the PI3K/AKT pathway that inhibits pGSK3, downregulating hyperphosphorylation in the mitochondria and thus preventing the formation of neurofibrillary tangles. Additionally, pAKT activates pCREB, increasing BDNF levels, which inhibit the neurotoxicity produced by neurofibrillary tangles and amyloid plaques. Abbreviations—p70s6k = phosphorylated p70 S6 kinase; IRS1 = insulin receptor substrate 1; MAPK = mitogen-activated protein kinase; mBDNF = mature brain-derived neurotrophic factor; mTORC1 = mammalian target of rapamycin complex 1; pAKT = phosphorylated RAC-alpha serine/threonine -protein kinase; pCREB = phosphorylated cAMP responsive element binding protein; pGSK = phosphorylated glycogen synthase kinase-3; PI3K = phosphatidylinositol 3-kinase; TrkB = tropomyosin receptor kinase B. This figure was created at https://app.biorender.com on 14 August 2023.

RET could mediate neuroprotection against AD through the activation of several signaling pathways during skeletal muscle activity. Upon skeletal muscle contractions, IGF1 binds to the IGF1 receptor (IGF1R), activating the insulin receptor 1 substrate within myocytes and subsequently activating PI3K. After PI3K activation, Akt is phosphorylated and modulates MAPK and ERK signaling, concluding with the phosphorylation, and thus the activation of CREB (pCREB), which is responsible for modulating BDNF messenger RNA (mRNA) transcription [95]. However, RET could also increase the activation of pCREB through the mTORC1 pathway, which would also activate BDNF mRNA transcription through pCREB and thus reduce NFTs and Aβ-mediated toxicity [96]. Mature BDNF (mBDNF) can be transported through the bloodstream until it reaches the brain, where it crosses the blood–brain barrier through a high-capacity saturable transport system [97]. In neurons, mBDNF binds to the tropomyosin kinase B receptor (TrkB). The results increase the activation of the PI3K/pAKT pathway. Subsequently, pAKT inhibits the phosphorylation of glycogen synthase kinase 3 (pGSK3), resulting in the inhibition of hyperphosphorylation in the mitochondria [98]. Furthermore, pAKT activates pCREB within the neuron, inducing an increase in BDNF levels in neurons, which could counteract the toxicity produced by both NFTs and Aβ plaques (Figure 3).

### 4.3. Resistance Exercise Training and Other Hypotheses for Alzheimer’s Disease

Regarding the vascular hypotheses, RET may act as a proinflammatory mediator in cardiometabolic diseases, including diabetes mellitus and arterial hypertension. Indeed, a systematic review with a meta-analysis and meta-regression of controlled trials evaluated the role of different training modalities and found that RET regulates some of the proinflammatory mediators [98]. The results suggest that all RET interventions are effective in reducing inflammatory status in subjects with cardiometabolic diseases, provided that the training leads to increases in maximal oxygen consumption (VO2 max), which is also enhanced with RET in older adults [99].

Regarding the oxidative hypothesis, RET can regulate proinflammatory factors as well as oxidative stress. The main protection is hypothesized to be exerted through redox homeostasis signaling by irisin, fibroblast growth factor 21 (FGF21), BDNF, and the lipid 12,13-diHOME [100]. Regarding the lipid hypothesis, RET can regulate changes in lipid metabolic pathways by gradually increasing the weight load, reducing the total cholesterol concentration, and increasing levels of high-density lipoprotein (HDL), LDL, and very low–density lipoprotein (VLDL) cholesterol [101]. Moreover, RET can regulate the blood metabolomic profile in humans, although its influence on the lipid metabolome remains unclear [102].

Regarding the neuroinflammation hypothesis, short-term RET improved cognitive function in 3xTg mice and conferred beneficial effects on neuroinflammation and amyloid and tau pathology, as well as synaptic plasticity [14]. RET decreased the amyloid load and modulated inflammatory responses in the APP/PS1 mouse model for AD [18]. RET could show a beneficial prophylactic effect on the cognitive deficiencies caused by acute neuroinflammation by increasing catalase activity [103]. Prehabilitive RET reduced neuroinflammation and improved mitochondrial health in aged mice with perioperative disorder [104]. RET modulated hippocampal neuroinflammation and protected against the anxiety–depression-like dyad induced with an emotional single prolonged stress model mouse [105]. Finally, IGF-1 may play a role in the mechanism behind the cognitive benefit of RET, and kynurenine may be a surrogate biomarker for neurodegeneration and cognitive decline [106]. RET is involved in the regulation of IL1-β by improving skeletal muscle strength and reducing fatigue in breast cancer survivors [107]. It has also been demonstrated that RET impacts IL-6 and cognitive function in older adults at risk of cognitive decline [108]. Furthermore, there has also been evidence of a decrease in IL-1 and TNF [109] as well as regulation of CXCL1 in a cell model [110], prostaglandins in humans [111], and ROS in young men [112].

## 5. Discussion and Conclusions

RET could play a significant role in the prevention of AD and even attenuate brain atrophy. However, a consensus must be reached on which variables are most relevant in preventing or attenuating AD, and whether to use muscle strength or muscle mass as the main outcome. It has been shown that body weight, muscle mass, and blood nutritional biomarkers improved in the intervention groups with RET compared to the control groups. However, there were no effects on cognition or physical outcomes [113]. The use of RET and even physical exercise in general is a hot research topic [114,115]. A meta-analysis showed consistently positive associations between the linear rate of change in grip strength and changes in cognitive functioning in older people [116], suggesting that gains in strength would be more relevant. On the other hand, improving muscular strength could enhance cognitive flexibility among older adults [117]. Further strengthening this hypothesis, in rats, RET could prevent and even reverse the skeletal muscle and brain atrophy associated with AD [118]. Moreover, RET reversed atrophy and reduced Aβ in animals [119]. RET can be divided based on the type of resistance exerted, which can be through integrated weight machines, free weights, or resistance bands. However, prescription is the most important aspect when planning a RET intervention. Prescription involves deciding the number of repetitions, the volume of work, and the rest periods [120]. Based on the current evidence, the most advisable exercise prescription is a workload of 70–80% of one-repetition maximum (1RM), with 8–12 repetitions, 3–5 sets per muscle group, rest for at least 2 min between sets, and training 2–3 times per week on non-consecutive days [120,121]. One of the future directions regarding RET is to determine the minimum amount of exercise required to achieve significant health benefits.

Regarding the effects of RET on BDNF, it is important to remember that there are two BDNF isoforms. pro-BDNF, the precursor of BDNF, is cleaved to produce mBDNF [122]. Acute RET has a more pronounced effect on increasing pro-BDNF rather than mBDNF in skeletal muscle, especially in type I fibers. However, additional comprehensive studies are necessary, because the increase in pro-BDNF or mBDNF may depend on the exercise intensity in humans [123]. In addition, the effects of chronic RET on pro-BDNF or mBDNF have not yet been studied. Moreover, although it has been demonstrated that BDNF can cross the blood–brain barrier through rapid and saturable transport, it also persisted in the brain and bloodstream after 60 min in an animal model [97]. The rate of force development could also serve as a useful indicator/biomarker of changes in neuromuscular function provoked by neurodegeneration [124]. Furthermore, researchers have recommended considering the involvement of caregivers in home-based exercise programs, as it could enhance the benefits of such programs for older adults with AD [125]. We have proposed the molecular mechanisms exerted by RET in patients with AD and highlighted interesting signaling pathways. Table 1 lists the studies we included in this review, which we have divided into three main categories: studies conducted on animals, studies conducted on cells, and studies conducted on humans.

Future directions include the incorporation of contemporary molecular biology techniques that allow for a more comprehensive characterization of the effects of RET on the prevention and attenuation of AD. A recommended protocol for RET for animal models such as rats is hypertrophic training, specifically climbing a vertical ladder with a height of 1.1 m at an 80° incline relative to the horizontal with extra weights tied to their tails [126]. Future studies could also add omics techniques such as genomics, transcriptomics, epigenetics, proteomics, and metabolomics, which could markedly enhance our understanding of how to regulate AD-associated markers through RET in humans [127]. Researchers could perform whole-genome analyses to assess the effects of RET on genes or genetic variants that have an impact on the pathophysiology of AD. Similarly, epigenetic studies could help to determine the effects of RET on AD by modulating methylation, microRNAs, and long non-coding RNAs [128,129]. Finally, with advances in bioinformatics, there are now more possibilities to study larger datasets, which could enrich research on the relationship between RET and AD.

This would enable the analysis of a greater amount of data or the characterizing of protein–protein interaction networks to identify key molecular targets [130]. This approach would also allow for the elimination of human bias in choosing molecular targets for experimental studies, thus optimizing both the time and resources allocated to research on RET and AD. By means of this method, different neuroplasticity pathways modulated by RET and aerobic training have been explored, determining multiple functional associations, highlighting in RET the production of IGF-1 and IGF-1R and the modulation of the Akt pathway [131]. In conclusion, there is clear evidence that RET could potentially prevent and even attenuate AD, evidencing a protection of brain structures affected by AD and cognitive benefits [132]. However, additional studies are needed to strengthen and confirm the hypotheses mentioned in this review. Ideally, translational studies are recommended to enhance our understanding of the molecular mechanisms in skeletal muscle and the brain in patients with AD. These studies should aim to transfer these results and extrapolate them to circulating samples in humans, allowing for a comprehensive understanding, while respecting the bioethical principles of molecular scientific research.

## Figures and Tables

**Table 1:** Summary of animal, cell, and human studies.

Species	Reference	Aim	Model/ Pathology
Animals	[14]	Determined whether short-term resistance exercise inhibits neuroinflammation and attenuated neuropathological changes in 3xTg-AD mice.	3xTg-AD mice
	[15]	Determined the potential protective effects of aerobic, resistance, and combined exercise methods on an AD-like model induced by ovariectomy and D-galactose administration.	AD-like model
	[17]	Determined the effects of aerobic training and resistance training on hippocampal BDNF and IGF-1 signaling, Aβ expression, and the myokine cathepsin B in the 3xTg-AD model.	3xTg-AD mice
	[18]	Analyzed hippocampal changes in Aβ load, inflammatory responses, and locomotor activity in a transgenic APP/PS1 mouse model of AD submitted to a resistance exercise program.	APP/PS1 mice
	[34]	Determined the mechanisms involved in overload-induced muscle hypertrophy in the context of type I diabetes, specifically examining whether such hypertrophy can counteract the muscle hypotrophy associated with the diabetic state.	Type I diabetes rats
	[69]	Determined the acute changes that occur in skeletal muscles following a single bout of high-resistance exercise and assessed the relationship between the acute molecular responses and long-term muscle hypertrophy induced by high-resistance exercise training.	Wistar rats
	[78]	Determined the molecular mechanism underlying the accelerated onset of AD induced by skeletal muscle atrophy.	5XFAD mice
	[79]	Determined the relationship between apolipoprotein E levels and Aβ accumulation in the brains of transgenic AβPPswe/tg2576 (tg2576) mice, a commonly used model for AD.	tg2576 mice
	[85]	Determined the impact of disease progression in AD on the physiological features of skeletal muscle using a mouse model.	3xTg-AD mice
	[89]	Determined the molecular mechanisms underlying the impairment of new protein synthesis in the synapse in AD pathology, particularly focusing on the Akt1/mTOR signaling pathways.	APP/PS1 mice
	[90]	Determined the therapeutic potential of the apolipoprotein E mimetic peptide COG1410 in AD using the transgenic APP/PS1 mouse model.	APP/PS1 mice
	[96]	Determined the effect of chronic endurance exercise on tau phosphorylation levels in the brain with an AD-like pathology, using a transgenic mouse model of tauopathies.	AD-like model
	[101]	Determined the effect of physical resistance training on lipid metabolism and apoptosis in the adult Wistar rat prostate.	Wistar rats
	[103]	Investigated the role of prophylactic muscular strength exercise in diminishing cognitive alterations and modifying antioxidant intracellular scenery in an animal neuroinflammatory model in the CA1 region of the hippocampus.	Wistar rats
	[104]	Evaluated the neuroprotective effect of preconditioning resistance training on aged mice undergoing abdominal surgery, and examined the underlying mechanisms related to the neuroinflammatory state and synaptic plasticity in the hippocampus.	C57BL/6N mice
	[105]	Evaluated the role of resistance exercise in mitigating anxiety and depression-like behaviors induced by stress in a mouse model. Specifically, the study investigated the effects of resistance exercise on behavioral phenotypes, hippocampal neuroinflammation, and the Akt/mTOR signaling pathway in mice subjected to a single prolonged emotional stress protocol.	Swiss mice
	[119]	Evaluated muscle cross-sectional area, myonuclear number, satellite cell content, and myosin heavy chain types in an animal model of AD, and examined the possible role of resistance training in controlling skeletal muscle size in this disease.	Wistar rat with induced AD
	[126]	Evaluated the effects of RET and/or glutamine supplementation on signaling protein synthesis in adult rat skeletal muscle.	Rats
Cells	[33]	Determined the molecular mechanisms underlying the cytotoxic effects of streptozotocin on HepG2 hepatoma cells, focusing specifically on the role of oxidative stress, mitochondrial dysfunction, and metabolic alterations.	HepG2 cells
	[36]	Determined the effect of streptozotocin on neuronal activity using a novel one-dimensional electro-plasmonic nanograting platform.	Neurons
	[53]	Determined the influence of cholesterol levels on the proteolytic processing of the amyloid precursor protein by the β-secretase Bace1 in living human neuroblastoma cells.	Neuroblastoma cells
	[64]	Determined the mechanism underlying the inefficient degradation of protein UBB (+1), which is generated from an erroneous transcriptional process of the ubiquitin B gene.	HeLa and SH-SY5Y cells
	[88]	Determined the impact of forebrain neuron exposure to suramin on the Akt/mTOR signaling pathway, a major regulator of autophagy, in comparison with rapamycin and chloroquine, and investigated the effect of suramin on several AD-related biomarkers in sporadic AD-derived forebrain neurons.	Neurons
	[92]	Determined the role of myelin breakdown in AD progression, focusing on its relation to oligodendrocyte progenitor cells and the neurotrophin system.	Oligodendrocytes
Humans	[7]	Examined the influence of age, education, and physical activity on executive function performance and the interaction effects between these factors on two subpopulations of adults, that is, young adults and middle-aged adults.	Healthy individuals
	[8]	Determined the impact of cognitive function on physical activity, physical function, and health-related quality of life in older adults within the first year after hip fracture surgery.	Healthy individuals
	[11]	Determined the hippocampal response to a 24-month physical activity intervention in sedentary older adults.	Healthy individuals
	[12]	Determined the effects of a long-term exercise intervention on two prominent biomarkers of inflammation (C-reactive protein and interleukin 6) in elderly men and women.	Healthy individuals
	[20]	Determined the incidence and distribution of various pathologies across neurodegenerative diseases and normal aging in a retrospective study of 1647 autopsied individuals.	Autopsied individuals
	[23]	Determined how frequently visual function deficits occur in the language variant of AD and how frequently language deficits occur in the visual variant of AD.	Patients with AD
	[24]	Determined the sex-specific differences and similarities in the hippocampus and its subfields (CA1 and CA3) in AD.	Autopsied individuals
	[26]	Developed an early and accurate diagnostic classification model for neurodegenerative dementia subtypes, specifically AD, frontotemporal dementia, and mild cognitive impairment, using resting-state functional magnetic resonance imaging data and clinical information.	Patients with AD
	[27]	Determined the relationship between baseline tau positron-emission tomography and the rate of subsequent clinical decline in individuals with atypical forms of early AD.	Patients with AD
	[36]	Determined the effect of a single bout of exercise on GLUT4 gene expression in muscle of patients with type 2 diabetes and control subjects, matched for age and body mass index.	Patients with type 2 diabetes
	[38]	Determined the relationship between aortic stiffening, as measured by aortic pulse wave velocity, and brain health indicators, specifically resting cerebral blood flow and cerebrovascular reactivity, in older adults.	Healthy individuals
	[39]	Examined temporal relationships between vascular stiffness, central hemodynamics, microvascular function, and blood pressure progression.	Patients with vascular injuries
	[84]	Examined body composition in individuals with early AD and without dementia and its relation to cognition and brain volume.	Patients with dementia
	[93]	Explored the relationship between BDNF and VEGF serum levels with future brain Aβ and tau pathology in a cohort of cognitively healthy, predominantly middle-aged adults, and tested for possible effect modifications by sex and menopausal status.	Healthy individuals
	[94]	Determined the potential of neurotrophin growth factors and their receptors as biomarkers for AD. Specifically, the researchers assessed the messenger RNA expression levels of neurotrophin growth factors (such as BDNF) and their receptors (such as NTRK2, TrkA, and TrkC) in blood samples collected from patients with AD and healthy controls.	Patients with AD
	[102]	Investigated and compared the molecular response to two different types of skeletal muscle activity, specifically endurance exercise and resistance exercise, using the untargeted metabolomics profiling of plasma.	Healthy individuals
	[106]	Observed the effect of 12 weeks of resistance training on peripheral biomarker levels, cognitive function changes, and their interrelationship in older adults with different risks of mild cognitive impairment. Specifically, the study investigated whether resistance training has differential effects on cognitive function and biomarker levels in older adults with a high risk of mild cognitive impairment compared with those with a low risk of mild cognitive impairment.	Older adults with a low and high risk of mild cognitive impairment
	[107]	Determined whether resistance training can improve inflammatory markers, fatigue (both sensations and fatigability), and physical performance in breast cancer survivors. Additionally, the study investigated whether changes in inflammatory markers, fatigue, and physical performance are associated with each other in this population.	Breast cancer survivors
	[109]	Evaluated the effects of resistance training combined with dietary advice on chronic inflammation in elderly individuals. Specifically, the study assessed changes in anthropometric parameters and inflammatory biomarkers before and after a long-term progressive resistance training program associated with dietary advice.	Elderly people
	[110]	Analyzed the impact of exercise on the serum of patients with advanced-stage pancreatic cancer and its effects on pancreatic cancer cell proliferation, motility, and apoptosis.	Patients with pancreatic cancer
	[111]	Determined the synergistic effects of gotu kola supplementation and multicomponent exercise on cognitive function, inflammation, and oxidative stress in older adults with mild cognitive impairment.	Patients with mild cognitive impairment
	[123]	Investigated the expression and regulation of BDNF and mature BDNF in human skeletal muscle and plasma under various physiological conditions, including rest, acute exercise, lactate infusion, and fasting.	Healthy individuals
	[127]	Characterized the genetic landscape of AD and related dementias through a two-stage genome-wide association study.	Patients with AD
	[128]	Investigated whether exercise training modifies the whole blood methylation profile in healthy women. Specifically, the study assessed changes in methylation patterns following a 14-week exercise training protocol, which included aerobic cardiorespiratory and muscle strength exercises.	Healthy individuals
	[129]	Investigated the modulation of microRNAs associated with myogenesis following physical performance, particularly in the context of running a half marathon.	Healthy individuals

Abbreviations—Aβ = amyloid beta; AD = Alzheimer’s disease; BDNF = brain-derived neurotrophic factor; GLUT4 = glucose transported 4; IGF-1 = insulin-like growth factor 1; mTOR = mammalian target of rapamycin; RET = resistance exercise training; VEGF = vascular endothelial growth factor.

## References

[1] Passeri E., Elkhoury K., Morsink M., Broersen K., Linder M., Tamayol A., Malaplate C., Yen F., Arab-Tehrany E. (2022). Alzheimer’s Disease: Treatment Strategies and Their Limitations. Int. J. Mol. Sci..

[2] McDade E., Llibre-Guerra J.J., Holtzman D.M., Morris J.C., Bateman R.J. (2021). The informed road map to prevention of Alzheimer Disease: A call to arms. Mol. Neurodegener..

[3] McKeown A., Turner A., Angehrn Z., Gove D., Ly A., Nordon C., Nelson M., Tochel C., Mittelstadt B., Keenan A. (2020). Health Outcome Prioritization in Alzheimer’s Disease: Understanding the Ethical Landscape. J. Alzheimer’s Dis..

[4] Zhu C.W., Sano M. (2006). Economic considerations in the management of Alzheimer’s disease. Clin. Interv. Aging.

[5] Kaur S., DasGupta G., Singh S. (2019). Altered Neurochemistry in Alzheimer’s Disease: Targeting Neurotransmitter Receptor Mechanisms and Therapeutic Strategy. Neurophysiology.

[6] Vasilopoulos F., Jeffrey H., Wu Y., Dumontheil I. (2023). Multi-level meta-analysis of whether fostering creativity during physical activity interventions increases their impact on cognitive and academic outcomes during childhood. Sci. Rep..

[7] Sharma N., Shenoy S. (2023). Role of Education and Physical Activity in Executive Function Performance of Adult Population. Curr. Aging Sci..

[8] Runde H.A., Taraldsen K., Follestad T., Saltvedt I., Johnsen L.G. (2023). The impact of cognitive function on physical activity, physical function and quality of life in older adults following a hip fracture. Age Ageing.

[9] Wu Y., Zang M., Wang B., Guo W. (2023). Does the combination of exercise and cognitive training improve working memory in older adults? A systematic review and meta-analysis. PeerJ.

[10] De Sá C.A., Saretto C.B., Cardoso A.M., Remor A., Breda C.O., da Silva Corralo V. (2023). Effects of a physical exercise or motor activity protocol on cognitive function, lipid profile, and BDNF levels in older adults with mild cognitive impairment. Mol. Cell. Biochem..

[11] Rosano C., Guralnik J., Pahor M., Glynn N.W., Newman A.B., Ibrahim T.S., Erickson K., Cohen R., Shaaban S.E., MacCloud R.L. (2017). Hippocampal Response to a 24-Month Physical Activity Intervention in Sedentary Older Adults. Am. J. Geriatr. Psychiatry.

[12] Nicklas B.J., Hsu F., Brinkley T.J., Church T., Goodpaster B.H., Kritchevsky S.B., Pahor M. (2008). Exercise Training and Plasma C-Reactive Protein and Interleukin-6 in Elderly People. J. Am. Geriatr. Soc..

[13] de Almeida E.J.R., Ibrahim H.J., Chitolina Schetinger M.R., de Andrade C.M., Cardoso A.M. (2022). Modulation of Inflammatory Mediators and Microglial Activation Through Physical Exercise in Alzheimer’s and Parkinson’s Diseases. Neurochem. Res..

[14] Liu Y., Chu J.M.T., Yan T., Zhang Y., Chen Y., Chang R.C.C., Wong G.T.C. (2020). Short-term resistance exercise inhibits neuroinflammation and attenuates neuropathological changes in 3xTg Alzheimer’s disease mice. J. Neuroinflam..

[15] Özbeyli D., Sarı G., Özkan N., Karademir B., Yüksel M., Çilingir Kaya Ö.T., Kasımay Çakır Ö. (2017). Protective effects of different exercise modalities in an Alzheimer’s disease-like model. Behav. Brain Res..

[16] Azevedo C.V., Hashiguchi D., Campos H.C., Figueiredo E.V., Otaviano S.F.S.D., Penitente A.R., Arida R.M., Longo B.M. (2023). The effects of resistance exercise on cognitive function, amyloidogenesis, and neuroinflammation in Alzheimer’s disease. Front. Neurosci..

[17] Pena G.S., Paez H.G., Johnson T.K., Halle J.L., Carzoli J.P., Visavadiya N.P., Zourdos M.C., Whitehurst M.A., Khamoui A.V. (2020). Hippocampal Growth Factor and Myokine Cathepsin B Expression following Aerobic and Resistance Training in 3xTg-AD Mice. Int. J. Chronic Dis..

[18] Hashiguchi D., Campos H.C., Wuo-Silva R., Faber J., Gomes da Silva S., Coppi A.A., Arida R.M., Longo B.M. (2020). Resistance Exercise Decreases Amyloid Load and Modulates Inflammatory Responses in the APP/PS1 Mouse Model for Alzheimer’s Disease. J. Alzheimer’s Dis..

[19] Fernandes J., Arida R.M., Gomez-Pinilla F. (2017). Physical exercise as an epigenetic modulator of brain plasticity and cognition. Neurosci. Biobehav. Rev..

[20] Robinson J.L., Xie S.X., Baer D.R., Suh E., Van Deerlin V.M., Loh N.J., Irwin D.J., McMillan C.T., Wolk D.A., Chen-Plotkin A. (2023). Pathological combinations in neurodegenerative disease are heterogeneous and disease-associated. Brain.

[21] Chatila Z.K., Bradshaw E.M. (2023). Alzheimer’s Disease Genetics: A Dampened Microglial Response?. Neuroscientist.

[22] Singh N.A., Sintini I. (2024). Editorial: New insights into atypical Alzheimer’s disease: From clinical phenotype to biomarkers. Front. Neurosci..

[23] Singh N.A., Graff-Radford J., Machulda M.M., Carlos A.F., Schwarz C.G., Senjem M.L., Jack C.R., Lowe V.J., Josephs K.A., Whitwell J.L. (2024). Atypical Alzheimer’s disease: New insights into an overlapping spectrum between the language and visual variants. J. Neurol..

[24] Onisiforou A., Christodoulou C.C., Zamba-Papanicolaou E., Zanos P., Georgiou P. (2024). Transcriptomic analysis reveals sex-specific patterns in the hippocampus in Alzheimer’s disease. Front. Endocrinol..

[25] Pelak V.S., Krishnan V., Serva S., Pressman P., Mahmood A., Noteboom L., Bettcher B.M., Sillau S.H., Callen A.L. (2024). Thaker, A.A. Lobar Microbleeds in the Posterior Cortical Atrophy Syndrome: A Comparison to Typical Alzheimer’s Disease. Curr. Neurol. Neurosci. Rep..

[26] Sadeghi M.A., Stevens D., Kundu S., Sanghera R., Dagher R., Yedavalli V., Jones C., Sair H., Luna L.P. (2024). Detecting Alzheimer’s Disease Stages and Frontotemporal Dementia in Time Courses of Resting-State fMRI Data Using a Machine Learning Approach. J. Imaging Inform. Med..

[27] Katsumi Y., Howe I.A., Eckbo R., Wong B., Quimby M., Hochberg D., McGinnis S.M., Putcha D., Wolk D., Touroutoglou A. (2024). Default mode network tau predicts future clinical decline in atypical early Alzheimer’s disease. medRxiv.

[28] Kametani F., Hasegawa M. (2018). Reconsideration of Amyloid Hypothesis and Tau Hypothesis in Alzheimer’s Disease. Front. Neurosci..

[29] Terry A.V., Buccafusco J.J. (2003). The Cholinergic Hypothesis of Age and Alzheimer’s Disease-Related Cognitive Deficits: Recent Challenges and Their Implications for Novel Drug Development. J. Pharmacol. Exp. Ther..

[30] Arnsten A.F.T., Datta D., Del Tredici K., Braak H. (2021). Hypothesis: Tau pathology is an initiating factor in sporadic Alzheimer’s disease. Alzheimer’s Dement..

[31] Arrué L., Cigna-Méndez A., Barbosa T., Borrego-Muñoz P., Struve-Villalobos S., Oviedo V., Martínez-García C., Sepúlveda-Lara A., Millán N., Márquez Montesinos J.C.E. (2022). New Drug Design Avenues Targeting Alzheimer’s Disease by Pharmacoinformatics-Aided Tools. Pharmaceutics.

[32] Figueroa P.B.S., Ferreira A.F.F., Britto L.R., Doussoulin A.P., Torrão A.d.S. (2021). Association between thyroid function and Alzheimer’s disease: A systematic review. Metab. Brain Dis..

[33] Raza H., John A. (2012). Streptozotocin-Induced Cytotoxicity, Oxidative Stress and Mitochondrial Dysfunction in Human Hepatoma HepG2 Cells. Int. J. Mol. Sci..

[34] Fortes M.A.S., Scervino M.V.M., Marzuca-Nassr G.N., Vitzel K.F., da Justa Pinheiro C.H., Curi R. (2017). Hypertrophy Stimulation at the Onset of Type I Diabetes Maintains the Soleus but Not the EDL Muscle Mass in Wistar Rats. Front. Physiol..

[35] Yao Q., Jiang K., Lin F., Zhu T., Khan N.H., Jiang E. (2023). Pathophysiological Association of Alzheimer’s Disease and Hypertension: A Clinical Concern for Elderly Population. Clin. Interv. Aging.

[36] Kadhim H.J., Al-Mumen H., Nahi H.H., Hamidi S.M. (2022). Streptozotocin-induced Alzheimer’s disease investigation by one-dimensional plasmonic grating chip. Sci. Rep..

[37] Hussey S.E., McGee S.L., Garnham A., McConell G.K., Hargreaves M. (2012). Exercise increases skeletal muscle GLUT4 gene expression in patients with type 2 diabetes. Diabetes Obes. Metab..

[38] Jefferson A.L., Cambronero F.E., Liu D., Moore E.E., Neal J.E., Terry J.G., Nair S., Pechman K.R., Rane S., Davis L.T. (2018). Higher Aortic Stiffness Is Related to Lower Cerebral Blood Flow and Preserved Cerebrovascular Reactivity in Older Adults. Circulation.

[39] Kaess B.M., Rong J., Larson M.G., Hamburg N.M., Vita J.A., Levy D., Benjamin E.J., Vasan R.S., Mitchell G.F. (2012). Aortic Stiffness, Blood Pressure Progression, and Incident Hypertension. JAMA.

[40] Watase H., Sun J., Hippe D.S., Balu N., Li F., Zhao X., Mani V., Fayad Z.A., Fuster V., Hatsukami T.S. (2018). Carotid Artery Remodeling Is Segment Specific. Arterioscler. Thromb. Vasc. Biol..

[41] Bajwa E., Klegeris A. (2022). Neuroinflammation as a mechanism linking hypertension with the increased risk of Alzheimer’s disease. Neural Regen. Res..

[42] Swerdlow R.H., Khan S.M. (2004). A “mitochondrial cascade hypothesis” for sporadic Alzheimer’s disease. Med. Hypotheses.

[43] Swerdlow R.H. (2018). Mitochondria and Mitochondrial Cascades in Alzheimer’s Disease. J. Alzheimer’s Dis..

[44] Song M., Fan X. (2023). Systemic Metabolism and Mitochondria in the Mechanism of Alzheimer’s Disease: Finding Potential Therapeutic Targets. Int. J. Mol. Sci..

[45] Swerdlow R.H. (2023). The Alzheimer’s Disease Mitochondrial Cascade Hypothesis: A Current Overview. J. Alzheimer’s Dis..

[46] Rose J., Brian C., Pappa A., Panayiotidis M.I., Franco R. (2020). Mitochondrial Metabolism in Astrocytes Regulates Brain Bioenergetics, Neurotransmission and Redox Balance. Front. Neurosci..

[47] Zhao R., Jiang S., Zhang L., Yu Z. (2019). Mitochondrial electron transport chain, ROS generation and uncoupling (Review). Int. J. Mol. Med..

[48] El-Osta H., Circu M.L. (2016). Mitochondrial ROS and Apoptosis. Mitochondrial Mechanisms of Degeneration and Repair in Parkinson’s Disease.

[49] Han Y., Liu D., Cheng Y., Ji Q., Liu M., Zhang B., Zhou S. (2023). Maintenance of mitochondrial homeostasis for Alzheimer’s disease: Strategies and challenges. Redox Biol..

[50] de Veij Mestdagh C.F., Smit A.B., Henning R.H., van Kesteren R.E. (2023). Mitochondrial Targeting against Alzheimer’s Disease: Lessons from Hibernation. Cells.

[51] Magistretti P.J., Allaman I. (2018). Lactate in the brain: From metabolic end-product to signalling molecule. Nat. Rev. Neurosci..

[52] Allinquant B., Clamagirand C., Potier M.C. (2014). Role of cholesterol metabolism in the pathogenesis of Alzheimer’s disease. Curr. Opin. Clin. Nutr. Metab. Care.

[53] Capitini C., Bigi A., Parenti N., Emanuele M., Bianchi N., Cascella R., Cecchi C., Maggi L., Annunziato F., Pavone F.S. (2023). APP and Bace1: Differential effect of cholesterol enrichment on processing and plasma membrane mobility. iScience.

[54] Windham I.A., Cohen S. (2024). The cell biology of APOE in the brain. Trends Cell Biol..

[55] Li D., Zhang J., Liu Q. (2022). Brain cell type-specific cholesterol metabolism and implications for learning and memory. Trends Neurosci..

[56] Zhou X., Shi Q., Zhang X., Gu L., Li J., Quan S., Zhao X., Li Q. (2023). ApoE4-mediated blood-brain barrier damage in Alzheimer’s disease: Progress and prospects. Brain Res. Bull..

[57] Akyol O., Akyol S., Chou M.C., Chen S., Liu C.K., Selek S., Soares J.C., Chen C.H. (2023). Lipids and lipoproteins may play a role in the neuropathology of Alzheimer’s disease. Front. Neurosci..

[58] Eikelenboom P., Veerhuis R. (1999). The importance of inflammatory mechanisms for the development of Alzheimer’s disease. Exp. Gerontol..

[59] Wong-Guerra M., Pardo-Andreu G.L., Nuñez-Figueredo Y. (2023). Modelos animales no transgénicos de demencia. consideraciones metodológicas y relevancia farmacológica. Rev. Cienc. Farm. Aliment..

[60] Morales I., GuzmÃ¡n-MartÃnez L., Cerda-Troncoso C., FarÃas G.A., Maccioni R.B. (2014). Neuroinflammation in the pathogenesis of Alzheimer’s disease. A rational framework for the search of novel therapeutic approaches. Front. Cell. Neurosci..

[61] Arias C., Sepúlveda P., Castillo R.L., Salazar L.A. (2023). Relationship between Hypoxic and Immune Pathways Activation in the Progression of Neuroinflammation: Role of HIF-1α and Th17 Cells. Int. J. Mol. Sci..

[62] DiSabato D.J., Quan N., Godbout J.P. (2016). Neuroinflammation: The devil is in the details. J. Neurochem..

[63] Di Benedetto S., Müller L., Wenger E., Düzel S., Pawelec G. (2017). Contribution of neuroinflammation and immunity to brain aging and the mitigating effects of physical and cognitive interventions. Neurosci. Biobehav. Rev..

[64] Verhoef L.G.G.C., Heinen C., Selivanova A., Halff E.F., Salomons F.A., Dantuma N.P. (2009). Minimal length requirement for proteasomal degradation of ubiquitin-dependent substrates. FASEB J..

[65] World Health Organization Global Action Plan on Physical Activity 2018–2030: More Active People for a Healthier World: At-A-Glance. 2018. https://iris.who.int/handle/10665/272721.

[66] Caspersen C.J., Powell K.E., Christenson G.M. (1985). Physical activity, exercise, and physical fitness: Definitions and distinctions for health-related research. Public Health Rep..

[67] Phillips S.M., Winett R.A. (2010). Uncomplicated Resistance Training and Health-Related Outcomes. Curr. Sports Med. Rep..

[68] Bodine S.C., Stitt T.N., Gonzalez M., Kline W.O., Stover G.L., Bauerlein R., Zlotchenko E., Scrimgeour A., Lawrence J.C., Glass D.J. (2001). Akt/mTOR pathway is a crucial regulator of skeletal muscle hypertrophy and can prevent muscle atrophy in vivo. Nat. Cell Biol..

[69] Baar K., Esser K. (1999). Phosphorylation of p70 ^S6k^ correlates with increased skeletal muscle mass following resistance exercise. Am. J. Physiol. Cell Physiol..

[70] Bolster D., Kubica N., Crozier S., Williamson D., Farrell P., Kimball S., Jefferson L.S. (2004). Understanding skeletal muscle hypertrophy: Integration of cell signalling. Physiol. News Mag..

[71] Glass D.J. (2005). Skeletal muscle hypertrophy and atrophy signaling pathways. Int. J. Biochem. Cell. Biol..

[72] Vainshtein A., Sandri M. (2020). Signaling Pathways That Control Muscle Mass. Int. J. Mol. Sci..

[73] Zanou N., Gailly P. (2013). Skeletal muscle hypertrophy and regeneration: Interplay between the myogenic regulatory factors (MRFs) and insulin-like growth factors (IGFs) pathways. Cell. Mol. Life Sci..

[74] Fernandes T., Soci Ú.P., Melo S.F., Alves C.R., Oliveira E.M. (2012). Signaling Pathways that Mediate Skeletal Muscle Hypertrophy: Effects of Exercise Training. Skeletal Muscle—From Myogenesis to Clinical Relations.

[75] Stone M.H., Collins D., Plisk S., Haff G., Stone M.E. (2000). Training Principles: Evaluation of Modes and Methods of Resistance Training. Strength Cond. J..

[76] Kasper K. (2019). Sports Training Principles. Curr. Sports Med. Rep..

[77] Wang J., Gu B.J., Masters C.L., Wang Y.J. (2017). A systemic view of Alzheimer disease—Insights from amyloid-β metabolism beyond the brain. Nat. Rev. Neurol..

[78] Nagase T., Tohda C. (2021). Skeletal muscle atrophy-induced hemopexin accelerates onset of cognitive impairment in Alzheimer’s disease. J. Cachexia Sarcopenia Muscle.

[79] Kuo Y.M., Crawford F., Mullan M., Kokjohn T.A., Emmerling M.R., Weller R.O., Roher A.E. (2000). Elevated Aβ and Apolipoprotein E in AβPP Transgenic Mice and Its Relationship to Amyloid Accumulation in Alzheimer’s Disease. Mol. Med..

[80] Waite S.J., Maitland S., Thomas A., Yarnall A.J. (2021). Sarcopenia and frailty in individuals with dementia: A systematic review. Arch. Gerontol. Geriatr..

[81] McKhann G., Drachman D., Folstein M., Katzman R., Price D., Stadlan E.M. (1984). Clinical diagnosis of Alzheimer’s disease. Neurology.

[82] Askanas V., Engel W.K. (2008). Inclusion-body myositis: Muscle-fiber molecular pathology and possible pathogenic significance of its similarity to Alzheimer’s and Parkinson’s disease brains. Acta Neuropathol..

[83] Uruha A., Nishino I. (2013). Pathogenesis of inclusion body myositis: Autoimmune or degenerative disease?. Brain Nerve.

[84] Burns J.M., Johnson D.K., Watts A., Swerdlow R.H., Brooks W.M. (2010). Reduced Lean Mass in Early Alzheimer Disease and Its Association With Brain Atrophy. Arch. Neurol..

[85] Monteiro-Cardoso V., Castro M., Oliveira M.M., Moreira P., Peixoto F., Videira R. (2015). Age-Dependent Biochemical Dysfunction in Skeletal Muscle of Triple- Transgenic Mouse Model of Alzheimer’s Disease. Curr. Alzheimer Res..

[86] Andrade L.J.d.O., Oliveira LMd Bittencourt A.M.V., Lourenço L.G.d.C., Oliveira G.C.M.d. (2024). Brain insulin resistance and Alzheimer’s disease: A systematic review. Dement Neuropsychol..

[87] Shafi O. (2016). Inverse relationship between Alzheimer’s disease and cancer, and other factors contributing to Alzheimer’s disease: A systematic review. BMC Neurol..

[88] Culibrk R.A., Ebbert K.A., Yeisley D.J., Chen R., Qureshi F.A., Hahn J., Hahn M.S. (2024). Impact of Suramin on Key Pathological Features of Sporadic Alzheimer’s Disease-Derived Forebrain Neurons. J. Alzheimer’s Dis..

[89] Kommaddi R.P., Gowaikar R., PA H., Diwakar L., Singh K., Mondal A. (2024). Akt activation ameliorates deficits in hippocampal-dependent memory and activity-dependent synaptic protein synthesis in an Alzheimer’s disease mouse model. J. Biol. Chem..

[90] Qiao Y., Liu H., He C., Ma Y. (2024). ApoE Mimic Peptide COG1410 Reduces Aβ Deposition and Improves Cognitive Function by Inducing the Transformation of A1/A2 Reactive Astrocytes and Increasing the BDNF Concentration in Brain of APP/PS1 Double Transgenic Mice. Neuroscience.

[91] Numakawa T., Kajihara R. (2024). An Interaction between Brain-Derived Neurotrophic Factor and Stress-Related Glucocorticoids in the Pathophysiology of Alzheimer’s Disease. Int. J. Mol. Sci..

[92] Zota I., Chanoumidou K., Charalampopoulos I., Gravanis A. (2024). Dynamics of myelin deficits in the 5xFAD mouse model for Alzheimer’s disease and the protective role of BDNF. Glia.

[93] Weinstein G., Kojis D.J., Ghosh S., Beiser A.S., Seshadri S. (2024). Association of Neurotrophic Factors at Midlife With In Vivo Measures of β-Amyloid and Tau Burden 15 Years Later in Dementia-Free Adults. Neurology.

[94] Asadi M.R., Gharesouran J., Sabaie H., Zaboli Mahdiabadi M., Mazhari S.A., Sharifi-Bonab M., Shirvani-Farsani Z., Taheri M., Sayad A., Rezazadeh M. (2024). Neurotrophin growth factors and their receptors as promising blood biomarkers for Alzheimer’s Disease: A gene expression analysis study. Mol. Biol. Rep..

[95] Gao L., Zhang Y., Sterling K., Song W. (2022). Brain-derived neurotrophic factor in Alzheimer’s disease and its pharmaceutical potential. Transl. Neurodegener..

[96] Leem Y.H., Lim H.J., Shim S.B., Cho J.Y., Kim B.S., Han P.L. (2009). Repression of tau hyperphosphorylation by chronic endurance exercise in aged transgenic mouse model of tauopathies. J. Neurosci. Res..

[97] Pan W., Banks W.A., Fasold M.B., Bluth J., Kastin A.J. (1998). Transport of brain-derived neurotrophic factor across the blood–brain barrier. Neuropharmacology.

[98] Del Rosso S., Baraquet M.L., Barale A., Defagó M.D., Tortosa F., Perovic N.R., Aoki M.P. (2023). Long-term effects of different exercise training modes on cytokines and adipokines in individuals with overweight/obesity and cardiometabolic diseases: A systematic review, meta-analysis, and meta-regression of randomized controlled trials. Obes. Rev..

[99] Kelley G.A., Kelley K.S., Stauffer B.L. (2024). Resistance training and inter-interindividual response differences on cardiorespiratory fitness in older adults: An ancillary meta-analysis of randomized controlled trials. Sci. Prog..

[100] Félix-Soriano E., Stanford K.I. (2023). Exerkines and redox homeostasis. Redox Biol..

[101] Teixeira G.R., Mendes L.O., Veras A.S.C., Thorpe H.H.A., Fávaro W.J., de Almeida Chuffa L.G., Pinheiro P.F.F., Martinez F.E. (2020). Physical resistance training-induced changes in lipids metabolism pathways and apoptosis in prostate. Lipids Health Dis..

[102] Morville T., Sahl R.E., Moritz T., Helge J.W., Clemmensen C. (2020). Plasma Metabolome Profiling of Resistance Exercise and Endurance Exercise in Humans. Cell. Rep..

[103] de Gregório E., Mendes G.C., Somensi L.B., Freire C.G., Lopes L.F., Lima K.R., Carrazoni G.S., Neves B.S., Picua S.S., da Silva L.M. (2022). Neuroprotective effects of strength training in a neuroinflammatory animal model. BMC Neurosci..

[104] Liu Y., Chu J.M.T., Ran Y., Zhang Y., Chang R.C.C., Wong G.T.C. (2022). Prehabilitative resistance exercise reduces neuroinflammation and improves mitochondrial health in aged mice with perioperative neurocognitive disorders. J. Neuroinflam..

[105] Jung J.T.K., Marques L.S., Zborowski V.A., Silva G.L., Nogueira C.W., Zeni G. (2023). Resistance Training Modulates Hippocampal Neuroinflammation and Protects Anxiety-Depression-like Dyad Induced by an Emotional Single Prolonged Stress Model. Mol. Neurobiol..

[106] Vints W.A.J., Gökçe E., Šeikinaitė J., Kušleikienė S., Česnaitienė V.J., Verbunt J., Levin J., Masiulis N. (2024). Resistance training’s impact on blood biomarkers and cognitive function in older adults with low and high risk of mild cognitive impairment: A randomized controlled trial. Eur. Rev. Aging Phys. Act..

[107] Martins F.M., Santagnello S.B., de Oliveira Junior G.N., de Sousa J.d.F.R., Michelin M.A., Nomelini R.S. (2023). Lower-Body Resistance Training Reduces Interleukin-1β and Transforming Growth Factor-β1 Levels and Fatigue and Increases Physical Performance in Breast Cancer Survivors. J. Strength Cond. Res..

[108] Alizaei Yousefabadi H., Niyazi A., Alaee S., Fathi M., Mohammad Rahimi G.R. (2021). Anti-Inflammatory Effects of Exercise on Metabolic Syndrome Patients: A Systematic Review and Meta-Analysis. Biol. Res. Nurs..

[109] Lopes L.M.P., Oliveira ECd Becker L.K., Costa G.d.P., Pinto K.M.d.C., Talvani A., Carraro J.C.C., Coelho D.B. (2020). Resistance Training Associated with Dietetic Advice Reduces Inflammatory Biomarkers in the Elderly. Biomed. Res. Int..

[110] Schwappacher R., Dieterich W., Reljic D., Pilarsky C., Mukhopadhyay D., Chang D.K., Biankin E.V., Siebler J., Herrmann H.J., Neurath M.F. (2021). Muscle-Derived Cytokines Reduce Growth, Viability and Migratory Activity of Pancreatic Cancer Cells. Cancers.

[111] Phoemsapthawee J., Ammawat W., Prasertsri P., Sathalalai P., Leelayuwat N. (2022). Does Gotu kola supplementation improve cognitive function, inflammation, and oxidative stress more than multicomponent exercise alone?—A randomized controlled study. J. Exerc. Rehabil..

[112] Jacko D., Masur L., Schaaf K., Zacher J., Bersiner K., de Marées M., Bloch W., Gehlert S. (2024). Resistance training does not increase myocellular garbage dumps: A pilot study on lipofuscin in skeletal muscle fibers of resistance trained young men. Physiol. Rep..

[113] Key M.N., Szabo-Reed A.N. (2023). Impact of Diet and Exercise Interventions on Cognition and Brain Health in Older Adults: A Narrative Review. Nutrients.

[114] Ayari S., Abellard A., Carayol M., Guedj É., Gavarry O. (2023). A systematic review of exercise modalities that reduce pro-inflammatory cytokines in humans and animals’ models with mild cognitive impairment or dementia. Exp. Gerontol..

[115] Alanazi M.A. (2024). The Role of Physical Activity in Adjunctive Nursing Management of Neuro-Degenerative Diseases among Older Adults: A Systematic Review of Interventional Studies. Life.

[116] Zammit A.R., Piccinin A.M., Duggan E.C., Koval A., Clouston S., Robitaille A., Brown C.L., Handschuh P., Wu C., Jarry V. (2021). A Coordinated Multi-study Analysis of the Longitudinal Association Between Handgrip Strength and Cognitive Function in Older Adults. J. Gerontol. Ser. B.

[117] García-Llorente A.M., Casimiro-Andújar A.J., Linhares D.G., De Souza Vale R.G., Marcos-Pardo P.J. (2024). Multidomain interventions for sarcopenia and cognitive flexibility in older adults for promoting healthy aging: A systematic review and meta-analysis of randomized controlled trials. Aging Clin. Exp. Res..

[118] Moss F.P., Leblond C.P. (1970). Nature of Dividing Nuclei in Skeletal Muscle of Growing Rats. J. Cell. Biol..

[119] Rahmati M., Shariatzadeh Joneydi M., Koyanagi A., Yang G., Ji B., Won Lee S., Keon Yon D., Smith L., Shin J.I., Yusheng L. (2023). Resistance training restores skeletal muscle atrophy and satellite cell content in an animal model of Alzheimer’s disease. Sci. Rep..

[120] Mcleod J.C., Currier B.S., Lowisz C.V., Phillips S.M. (2024). The influence of resistance exercise training prescription variables on skeletal muscle mass, strength, and physical function in healthy adults: An umbrella review. J. Sport Health Sci..

[121] Currier B.S., Mcleod J.C., Banfield L., Beyene J., Welton N.J., D’Souza A.C., Keogh J.A.J., Lin L., Coletta G., Yang A. (2023). Resistance training prescription for muscle strength and hypertrophy in healthy adults: A systematic review and Bayesian network meta-analysis. Br. J. Sports Med..

[122] Foltran R.B., Diaz S.L. (2016). BDNF isoforms: A round trip ticket between neurogenesis and serotonin?. J. Neurochem..

[123] Edman S., Horwath O., Van der Stede T., Blackwood S.J., Moberg I., Strömlind H., Nordström F., Ekblom M., Katz A., Apró W. (2024). Pro-Brain-Derived Neurotrophic Factor (BDNF), but Not Mature BDNF, Is Expressed in Human Skeletal Muscle: Implications for Exercise-Induced Neuroplasticity. Function.

[124] Lomborg S.D., Dalgas U., Hvid L.G. (2022). The importance of neuromuscular rate of force development for physical function in aging and common neurodegenerative disorders—A systematic review. J. Musculoskelet. Neuronal Interact.

[125] Braz de Oliveira M.P., Moreira Padovez R.d.F.C., Serrão P.R.M.d.S., de Noronha M.A., Cezar N.O.d.C., Andrade L.P.d. (2023). Effectiveness of physical exercise at improving functional capacity in older adults living with Alzheimer’s disease: A systematic review of randomized controlled trials. Disabil. Rehabil..

[126] Rodrigues Junior C.F., Murata G.M., Gerlinger-Romero F., Nachbar R.T., Marzuca-Nassr G.N., Gorjão R., Vitzel K.F., Hirabara S.M., Pithon-Curi T.C., Curi R. (2023). Changes in Skeletal Muscle Protein Metabolism Signaling Induced by Glutamine Supplementation and Exercise. Nutrients.

[127] Bellenguez C., Küçükali F., Jansen I.E., Kleineidam L., Moreno-Grau S., Amin N., Naj A.C., Campos-Martin R., Grenier-Boley B., Andrade V. (2022). New insights into the genetic etiology of Alzheimer’s disease and related dementias. Nat. Genet..

[128] da Silva Rodrigues G., Noronha N.Y., Almeida M.L., Sobrinho A.C.d.S., Watanabe L.M., Pinhel M.A.d.S., de Lima J.G.R., Zhang R., Nonino C.B., Alves C.R.R. (2023). Exercise training modifies the whole blood DNA methylation profile in middle-aged and older women. J. Appl. Physiol..

[129] Dalle Carbonare L., Dorelli G., Li Vigni V., Minoia A., Bertacco J., Cheri S., Deiana M., Innamorati G., Cominacini M., Tarperi C. (2022). Physical Activity Modulates miRNAs Levels and Enhances MYOD Expression in Myoblasts. Stem Cell Rev. Rep..

[130] Ramírez D., Caballero J. (2016). Is It Reliable to Use Common Molecular Docking Methods for Comparing the Binding Affinities of Enantiomer Pairs for Their Protein Target?. Int. J. Mol. Sci..

[131] Coutinho L.A., Leão L.L., Cassilhas R.C., de Paula A.M.B., Deslandes A.C., Monteiro-Junior R.S. (2022). Alzheimer’s disease genes and proteins associated with resistance and aerobic training: An in silico analysis. Exp. Gerontol..

[132] Nicola L., Loo S.J.Q., Lyon G., Turknett J., Wood T.R. (2024). Does resistance training in older adults lead to structural brain changes associated with a lower risk of Alzheimer’s dementia? A narrative review. Ageing Res. Rev..

